# Cardiometabolic Risk Parameters in “Nonfunctioning” Adrenal Incidentalomas: A Systematic Review and Meta-analysis

**DOI:** 10.1210/clinem/dgaf597

**Published:** 2025-10-30

**Authors:** Vittoria Favero, Davide Bernasconi, Alessandro Maloberti, Chiara Parazzoli, Antonio Musolino, Robert Chilton, Anthony P Heaney, Cristina Eller-Vainicher, Alfredo Scillitani, Iacopo Chiodini

**Affiliations:** Department of Medical Biotechnology and Translational Medicine, University of Milan, 20100 Milan, Italy; Unit of Endocrinology, ASST Grande Ospedale Metropolitano Niguarda, 20163 Milan, Italy; Bicocca Bioinformatics Biostatistics and Bioimaging B4 Center, Department of Medicine and Surgery, University of Milano-Bicocca, 20126 Monza, Italy; Unit of Clinical Research and Innovation, ASST Grande Ospedale Metropolitano Niguarda, 20163 Milan, Italy; School of Medicine and Surgery, University of Milano-Bicocca, 20126 Milan, Italy; Unit of Cardiology-Diagnostic and Rehabilitative, ASST Grande Ospedale Metropolitano Niguarda, 20163 Milan, Italy; Department of Medical Biotechnology and Translational Medicine, University of Milan, 20100 Milan, Italy; Unit of Endocrinology, ASST Grande Ospedale Metropolitano Niguarda, 20163 Milan, Italy; Department of Medical Biotechnology and Translational Medicine, University of Milan, 20100 Milan, Italy; Division of Cardiology, University of Texas Health Science Center - San Antonio, San Antonio, TX 78299, USA; Department of Medicine, Endocrinology, University of California, Los Angeles, Los Angeles, CA 90095, USA; Unit of Endocrinology, Fondazione IRCCS Cà Granda Ospedale Maggiore Policlinico, 20122 Milan, Italy; Unit of Endocrinology, Ospedale “Casa Sollievo Della Sofferenza”, 71013 Foggia, Italy; Department of Medical Biotechnology and Translational Medicine, University of Milan, 20100 Milan, Italy; Unit of Endocrinology, ASST Grande Ospedale Metropolitano Niguarda, 20163 Milan, Italy

**Keywords:** adrenal incidentalomas, intima-media thickness, insulin resistance, HOMA-IR, cortisol

## Abstract

**Objective:**

Several studies have investigated whether patients with nonfunctioning adrenal incidentalomas (NFAI) have an increased cardiometabolic risk, based on surrogate parameters such as the augmentation index (Aix), carotid intima-media thickness (cIMT), flow-mediated dilation (FMD), insulin resistance (IR), left ventricular mass index (LVMI), and pulse wave velocity (PWV), compared to patients without adrenal incidentalomas (AI). We sought to analyze the available literature to evaluate AIx, cIMT, FMD, IR, LVMI, and PWV in patients with NFAI as compared to patients without AI.

**Design:**

Systematic review and meta-analysis.

**Methods:**

We included studies that evaluated the cIMT and IR (primary outcomes) and AIx, FMD, LVMI, and PWV (secondary outcomes) in patients with NFAI vs matched subjects without AI. A random-effects model (DerSimonian and Laird) was used to calculate the standardized mean difference and 95% CI for each outcome.

**Results:**

Among the 24 available studies, 21 studies provided the necessary data (2228 subjects, mean age 53.3 ± 2.9 years, 35% males). Data on cIMT, IR, AIx, FMD, LVMI, and PWV were reported in 12 (1063 subjects), 15 (1745 subjects), 2 (140 subjects), 3 (198 subjects), 3 (201 subjects), and 2 (140 subjects) studies, respectively. Compared with patients without AI, patients with NFAI showed statistically significant differences in cIMT, IR, AIx, FMD, LVMI, and PWV (standardized mean difference and 95% CI: 1.22, 0.87-1.58; 0.51, 0.32-0.69; 0.94, 0.38-1.50; -1.24, -1.84- -0.65; 0.41, 0.04-0.78; 1.03, 0.16-1.89, respectively).

**Conclusion:**

Based on cIMT, IR, AIx, FMD, LVMI, and PWV levels, patients with NFAI may be at increased cardiovascular risk

Incidentally discovered adrenal masses, known as adrenal incidentalomas (AI), are relatively common in the general population. Among individuals older then age 60 years, the estimated prevalence of AI is 7% (1). Approximately one half of patients with AI may present with mild autonomous cortisol secretion (MACS), characterized by biochemical hypercortisolism (ie, cortisol levels after 1 mg overnight dexamethasone suppression test [F-1mgDST], above 1.8 µg/dL [50 nmol/L]) in the absence of the classical signs and symptoms of overt cortisol excess (2). Though asymptomatic, patients with MACS are at increased risk of cardiometabolic comorbidities, cardiovascular events, bone fragility, and, ultimately, mortality (3-5). Given its not-negligible prevalence and important clinical consequences (3, 4, 6, 7), recognizing MACS is important, especially given that MACS-related comorbidities improve after adrenalectomy (3, 4, 6, 7).

Recent evidence suggests that patients with so-called “nonfunctioning” adrenal incidentalomas (NFAIs), defined by post-F-1mgDST cortisol levels <1.8 µg/dL (50 nmol/L), may nonetheless be at increased risk of cardiometabolic complications and bone fragility compared to individuals without adrenal lesions (8-11). Importantly, in a subset of these patients, adrenalectomy has resulted in postoperative adrenal insufficiency, indicating the presence of subtle autonomous cortisol secretion not detected by the current F-1mgDST threshold of 1.8 µg/dL (50 nmol/L) (12). Additionally, the increased mortality suggested in patients with NFAI (13), comparable to that reported in individuals with MACS (14), further supports the hypothesis that a degree of clinically relevant cortisol excess may be present in at least a proportion of patients classified as having NFAI. Given the accumulating evidence, the presence of increased cardiometabolic risk in patients with NFAI is currently a topic of considerable debate and the appropriateness of the currently adopted F-1mgDST threshold of 1.8 µg/dL (50 nmol/L) is being questioned (15).

Today, several parameters, such as the augmentation index (AIx), carotid intima-media thickness (cIMT), flow-mediated dilation (FMD), insulin resistance, left ventricular mass index (LVMI), and pulse wave velocity (PWV) are considered reliable indexes of cardiometabolic risk (16-18). In particular, the cIMT (ie, thickness of the intimal and medial layers of the carotid artery wall) is a widely recognized marker of subclinical atherosclerosis (19) and a reliable indicator of cardiovascular risk (20). Similarly, the Homeostasis Model Assessment for Insulin Resistance (HOMA-IR) is used to measure insulin resistance in large population-based studies (21) and has been shown to predict the risk of diabetes, hypertension, and nonfatal major adverse cardiovascular events (22).

Several authors have investigated AIx, cIMT, FMD, HOMA-IR, LVMI, and PWV in patients with NFAI compared with individuals without adrenal lesions. These have yield inconclusive results, likely because of the limited sample sizes of the individual studies (23-43).

Therefore, the aim of the present study was to systematically review the available evidence and conduct a meta-analysis to determine whether patients with NFAI exhibit differences in cIMT and HOMA-IR (primary outcomes) and in FMD, LVMI, and PWV (secondary outcomes) compared to individuals without adrenal lesions.

## Methods

This study (PROSPERO: https://www.crd.york.ac.uk/PROSPERO/view/CRD420251069524), has been performed following the Preferred Reporting Items for Systematic reviews and Meta-Analyses statement (44).

### Search Strategy

Two reviewers (V.F. and I.C.) independently screened titles and abstracts and examined the full text of potentially relevant studies. Discordances were resolved by a third reviewer (A.S.).

**Figure 1.**
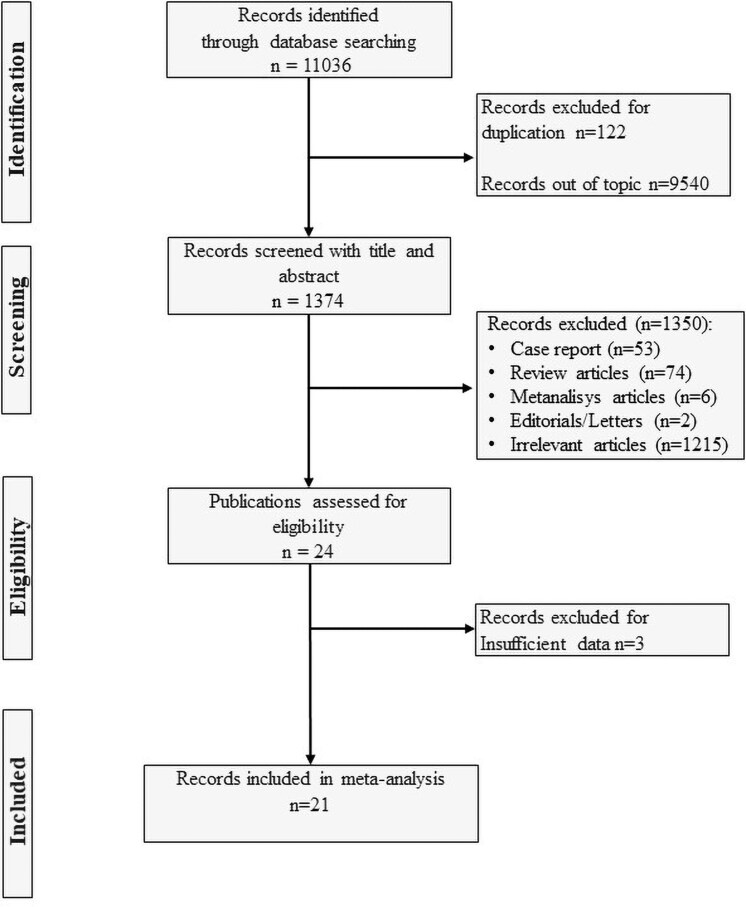
Study selection process. Potentially relevant studies. Discordances were resolved by a third reviewer (A.S.). Web of Science, Scopus, and PubMed were searched between January 1990 and May 2025 using the following keywords and medical subject headings: “non-functioning adrenal incidentalomas, non-functioning adrenal adenomas, mild autonomous cortisol secretion, adrenal adenomas, adrenal incidentalomas, hidden hypercortisolism, occult hypercortisolism, subclinical hypercortisolism, subclinical Cushing's syndrome, mild hypercortisolism, less severe hypercortisolism” (Fig. 1). A further analysis of the reference lists of the eligible studies was implemented to find out other additional publications. The Mendeley Desktop application (version 2.112.0, Mendeley Ltd) was used to remove duplicates and apply the inclusion criteria.

Web of Science, Scopus, and PubMed were searched between January 1990 and May 2025 using the following keywords and medical subject headings: “non-functioning adrenal incidentalomas, non-functioning adrenal adenomas, mild autonomous cortisol secretion, adrenal adenomas, adrenal incidentalomas, hidden hypercortisolism, occult hypercortisolism, subclinical hypercortisolism, subclinical Cushing's syndrome, mild hypercortisolism, less severe hypercortisolism” (Fig. 1). A further analysis of the reference lists of the eligible studies was implemented to find additional publications.

The Mendeley Desktop application (version 2.134.0, Mendeley Ltd) was used to remove duplicates and apply the inclusion criteria.

### Study's Selection

Only case-control studies (observational retrospective or prospective) including subjects ≥18 years of age with NFAI and control subjects without adrenal lesion were included. We included studies basing the diagnosis of NFAI on the criteria suggested by the European Society Guidelines (45), which are: (1) an incidentally discovered adrenal mass >1 cm in size diagnosed by imaging (computed tomography or magnetic nuclear resonance) performed for unrelated disorder; (2) absence of signs or symptoms of hypercortisolism; (3) no exogenous glucocorticoids administration during the past 3 months; and (4) F-1mgDST levels ≤1.8 μg/dL (50 nmol/L).

We excluded: (1) studies reporting inclusion of patients with NFAI but failing to exclude those with a F-1mgDST >1.8 µg/dL or using alternative criteria to rule out cortisol hypersecretion; (2) case reports and case series; (3) studies involving patients with NFAI without a matched control group of subjects without adrenal lesions, as confirmed by imaging studies; (4) studies including patients with AI presenting hypersecretion of catecholamines, sex hormones, or mineralocorticoids; and (5) studies involving patients with adrenocortical carcinomas or adrenal metastases (Fig. 1).

### Study Outcomes and Data Extraction

In this systematic review, outcomes were classified as primary or secondary based on the number of studies reporting each outcome: those reported in 5 or more studies were considered primary outcomes, whereas those reported in fewer than 5 studies were considered secondary outcomes. The predefined primary outcomes were the cIMT and HOMA-IR levels in patients with NFAI compared to controls without AI. We considered valid the assessment of cIMT and HOMA-IR according to the current clinical practice recommendations (21, 46). The secondary outcomes were AIx, FMD, LVMI, and PWV levels as determined in accordance with the commonly used methods (17, 18, 47-49).

Each study was searched by 2 authors (V.F. and I.C.). The following data have been obtained from patients and controls (if available): authors and study location, period of time, patients’ ethnicity, study design, sample size, mean age, percentage of male patients, outcome type (ie, AIx, cIMT, FMD, LVMI, HOMA-IR, and PWV), F-1mgDST mean ± SD or median levels with interquartile range, matching criteria (ie, age, sex, body mass index [BMI], smoking status, and alcohol use) and presence of significant comorbidities of the included subjects (ie, type 2 diabetes, hypertension, cardiovascular disease, dyslipidemia).

### Quality Assessment

Two investigators (V.F. and I.C.) assessed both the quality of the individual studies and the overall quality of evidence. The modified Newcastle–Ottawa Scale was used to assess the quality of the studies (www.ohri.ca/programs/clinical_epidemiology/oxford.asp).

We evaluated the following items: study sample, selection criteria of patients with NFAI (based on F-1mgDST), comparability (whether patients with NFAI and controls were matched for age, sex, BMI, and smoking habit), and outcome definitions (ie, how AIx, cIMT, FMD, HOMA-IR, LVMI, and PWV were assessed).

### Data Synthesis and Statistical Analysis

We conducted a meta-analysis of all eligible studies and obtained the pooled estimate in patients with NFAI compared to patients without NFAI separately for cIMT and HOMA-IR (primary outcomes) and for AIx, FMD, LVMI, and PWV (secondary outcomes). All analyses were performed with R version 4.0.3 (R Foundation for Statistical Computing, Vienna, Austria) and, in particular, with the package “meta” (50). Random-effects meta-analysis has been performed by first deriving an estimate of the between-study variation and heterogeneity. Subsequently, these results have been used for combining results (ie, for estimating the effect) and for developing the figure of primary interest.

The DerSimonian and Laird method, the conventionally used approach for random effects meta-analysis (51), has been used for calculating the association estimates (standardized mean difference [SMD]) and their 95% CI. The heterogeneity between studies was quantified using *I*^2^ and τ^2^ statistics. For the primary outcomes (cIMT and HOMA-IR), we conducted an analysis using Funnel Plots and Egger Test to evaluate the presence of possible publication bias and implemented an influence analysis with the leave-one-out method (omitting 1 study at a time) to investigate the impact of each study-specific association estimate on the pooled SMD. Finally, only for primary outcomes, we conducted a meta-regression analysis to assess the impact of several covariates (mean or median age, proportion of males, mean or median BMI, and proportion of smokers) on the SMD, accounting also for the size of each study. A *P* value < .05 was considered statistically significant.

## Results

### Study Selection Process

The study selection process is summarized in Fig. 1. We identified 11 036 studies from the different searched databases and excluded 122 for duplication and 9540 studies for lack of relevance. The remaining 1374 studies were first screened by reading the title and abstract. All studies reporting data on the AIx, cIMT, FMD, HOMA-IR, LVMI, and PWV values of NFAI patients and of matched subjects without adrenal lesions used as controls were evaluated for inclusion (n = 24). We excluded 1350 studies as they were meta-analysis articles (n = 6), case reports (n = 53), review articles (n = 74), editorial or letters (n = 2), or because they were not relevant for the aims of the present meta-analysis (n = 1215). Among the 24 publications assessed for eligibility, 3 studies were excluded because the values of the outcomes were not reported (52, 53) or because the mean F-1mgDST levels in NFAI were >1.8 µg/dL (50 nmol/L) (54) as summarized in Table 1. The interrater reliability between the 2 authors in the selection process was strong (κ = 0.89).

**Table 1.** Summary of the main characteristics of the excluded studies

Author	Country	Sample (n)	Main results and reasons for exclusion
Anderwald et al. 2013 (52)	Austria	180	Lower insulin sensitivity in NFAI patients than in controls; cIMT and HOMA-IR values not reported
Babinska et al. 2018 (54)	Poland	36	Higher HOMA-IR values in NFAI patients than in controls. Mean F-1mgDST levels in NFAI >1.8 µg/dL
Parasiliti-Caprino et al. 2024 (53)	Italy	1997	Increased cardiometabolic risk in NFAI patients than in controls. CIMT and HOMA-IR values not reported

Abbreviations: cIMT, carotid intima-media thickness; F-1mgDST, cortisol levels after 1 mg overnight dexamethasone suppression test; HOMA-IR, Homeostasis Model Assessment for Insulin Resistance; LV, left ventricular; NFAI, nonfunctioning adrenal incidentaloma.

### Studies Characteristics

The characteristics of the 21 studies that were used in the meta-analysis (23-43) are reported in Table 2. The 21 included studies provided cross-sectional data from 2228 subjects, mean age 53.3 ± 2.9 years, 35% males, and mean BMI 29.1 ± 2.0 kg/m^2^. Data collection was prospective and retrospective in 13 and 2 studies, respectively, whereas the study design was not reported in 6 studies. The quality of included studies varied among the included studies (Newcastle-Ottawa Scale between 4 and 7, with 20 of 21 studies ranging between 5 and 7).

**Table 2.** Summary of characteristics and quality evaluation by Newcastle Ottawa Scale score (NOS) of the studies included in the metanalysis

Author and reference	Country	Design	Size (n)	Age (y)	BMI (kg/m)	Males (%)	AIx/CIMT/FMD/IR/LVMI/PWV	Excl DM/AH/DL smoke/ CVE	Matching variables	NOS (0-9)
Ermetici et al. 2008 (24)	Italy	Prosp.	39	55.3	30.9	33	No/no/no/yes/yes/no	No/no/no/no/no	Gender, BMI	5
Yener et al. 2009 (25)	Turkey	Prosp.	116	51.8	28.3	27	No/yes/no/yes/no/no	Yes/yes/yes/yes/yes	Age, gender, BMI	6
Erbil et al. 2009 (42)	Turkey	n.s.	70	49.1	29.5	9	No/no/yes/yes/no/no	No/no/no/no/no	Age, gender, BMI, CVE, DM	7
Yener et al. 2011 (27)	Turkey	Prosp.	102	51.6	27.1	77	No/yes/yes/yes/no/no	Yes/no/no/no/yes	Age, gender, BMI	5
Arduc et al. 2014 (30)	Turkey	Prosp.	265	50.1	29.0	22	No/no/no/yes/no/no	No/no/no/no/no	Age, gender, BMI	6
Tuna et al. 2014 (29)	Turkey	n.s.	69	50.5	30.1	27	No/yes/no/no/no/no	Yes/no/yes/yes/no	Age, gender, BMI	5
Androulakis et al. 2014 (28)	Greece	Prosp.	265	53.9	26.5	41	No/yes/yes/yes/no/no	Yes/yes/yes/yes/yes	Age, gender, BMI	7
Delibasi et al. 2015 (41)	Turkey	Prosp.	75	53.7	28.1	30	No/yes/no/yes/no/no	Yes/no/no/no/yes	Age, gender, BMI	6
Evran et al. 2016 (31)	Turkey	Prosp.	109	51.1	28.4	39	No/yes/no/yes/no/no	Yes/yes/yes/yes/yes	Age, gender, BMI	7
Imga et al. 2016 (32)	Turkey	Prosp	86	53.0	30.6	24	No/yes/no/yes/yes/no	Yes/yes/yes/yes/yes	Age, gender, BMI	7
Akkan et al. 2017 (40)	Turkey	Prosp	70	51.0	29.5	42	Yes/no/no/no/no/yes	Yes/yes/yes/yes/yes	Age, gender, BMI	7
Cansu et al. 2017 (33)	Turkey	Prosp	70	52.1	28.3	31	Yes/yes/no/yes/no/yes	Yes/yes/yes/yes/yes	Age, gender, BMI	7
Kizilgul et al. 2017 (34)	Turkey	n.s.	68	52.8	29.7	43	No/yes/no/no/no/no	No/no/no/no/no	Gender, BMI	4
Sokmen et al. 2018 (43)	Turkey	n.s.	76	51.2	33.2	13	No/no/no/no/yes/no	No/no/no/no/yes	Age, gender	4
Marina et al. 2018 (35)	Serbia	Prosp.	57	62.0	27.0	0	No/no/no/yes/no/no	Yes/no/no/no/no	Age, gender, BMI	5
Emral et al. 2019 (36)	Turkey	n.s.	139	54.4	29.7	36	No/no/no/yes/no/no	Yes/no/no/no/yes	Age, gender, BMI	5
Peppa et al. 2010 (26)	Greece	Prosp.	66	52.9	29.6	67	No/no/no/yes/no/no	Yes/yes/yes/yes/yes	Age, gender, BMI	7
Kim et al. 2020 (37)	Korea	Retrosp.	320	55.7	24.2	73	No/no/no/yes/no/no	No/no/no/no/no	Age, gender, BMI, Smoke	6
Karatas et al. 2023 (23)	Turkey	Retrosp.	202	53.6	32.2	33	No/no/no/yes/no/no	No/no/no/no/no	Age, gender, BMI	6
Szychlińska et al. 2023 (38)	Poland	n.s.	92	57.8	27.8	42	No/no/no/no/no/yes	Yes/no/no/no/yes	Age, gender, BMI	5
Boyraz et al. 2025 (39)	Turkey	Prosp.	160	55.5	31.1	20	Yes/no/no/no/no/no	No/no/no/no/yes	Age, gender, BMI	5

Abbreviations: AH, arterial hypertension; Aix, augmentation index; BMI, body mass index; cIMT, carotid intima-media thickness; CVE, cardiovascular events; DL, dyslipidemia; DM, diabetes mellitus; Excl., excluded; FMD, flow-mediated dilation; HOMA-IR, Homeostasis Model Assessment for Insulin Resistance; LVMI, left ventricular mass index; n.s., not specified; NOS, Newcastle-Ottawa Scale; Prosp, prospective; PWV, pulse wave velocity; Retrosp, retrospective.

Regarding the primary outcomes, in 12 studies regarding overall 1063 subjects (592 patients with NFAI and 471 controls), cIMT values were reported (25, 27-29, 31-34, 36, 38, 39, 41). Among these studies, type 2 diabetes, arterial hypertension, dyslipidemia, smoking habit, and a history of cardiovascular events represented an exclusion criterion in 10, 5, 7, 6, and 10 cases, respectively. The HOMA-IR values were available from 15 studies (23-28, 30-33, 35-38, 41), including a total of 1715 subjects (841 patients with NFAI and 874 controls). In all studies, the presence of primary aldosteronism has been excluded.

Regarding the secondary outcomes, the AIx values were available from 2 studies (33, 40) including a total of 140 subjects (70 patients with NFAI and 70 controls), the FMD values were available from 3 studies (27, 28, 42) including a total of 198 subjects (109 patients with NFAI and 89 controls), the LVMI values were available from 3 studies (24, 32, 43) including a total of 201 subjects (102 patients with NFAI and 99 controls), and the PWV values were available from 2 studies (33, 40) including a total of 140 subjects (70 patients with NFAI and 70 controls).

The geographic areas of the included studies were Europe (n = 20) and East Asia (n = 1). Among these studies, type 2 diabetes, arterial hypertension, dyslipidemia, smoking habit, and a history of cardiovascular events represented an exclusion criterion in 13, 7, 8, 8, and 13 cases, respectively.

In all studies, the control group included subjects in whom the presence of adrenal lesions was excluded by appropriate imaging. Control groups were gender and BMI matched in 3 studies (24, 34, 43); age, gender, and BMI matched in 16 studies (23, 25-33, 35-43); and age, gender, BMI, and smoking habit matched in 2 studies (26, 37).

### Comparison of cIMT and HOMA-IR (Primary Outcomes) Values Between Patients With NFAIs and Controls

The number of included studies, sample size considered, and the SMD with 95% CI of cIMT and HOMA-IR between patients and controls are reported in Table 3.

**Table 3.** Number of included studies, sample size considered and standardized mean difference with 95% confidence interval between patients and controls for each specific outcome

Outcome	Included studies n	Total subjects n	Cases n	Controls n	SMD	95% CI
CIMT	12	1063	592	471	1.22	0.87-1.58
HOMA-IR	15	1715	841	874	0.51	0.32-0.69
AIx	2	140	70	70	0.94	0.38-1.50
FMD	3	198	109	89	-1.24	-1.84 to -0.65
LVMI	3	201	102	99	0.41	0.04-0.78
PWV	2	140	70	70	1.03	0.16-1.89

Abbreviations: Aix, augmentation index; cIMT, carotid intima-media thickness; FMD, flow-mediated dilation; HOMA-IR, Homeostasis Model Assessment for Insulin Resistance; LVMI, left ventricular mass index; PWV, pulse wave velocity; SMD, standardized mean difference.

**Figure 2.**
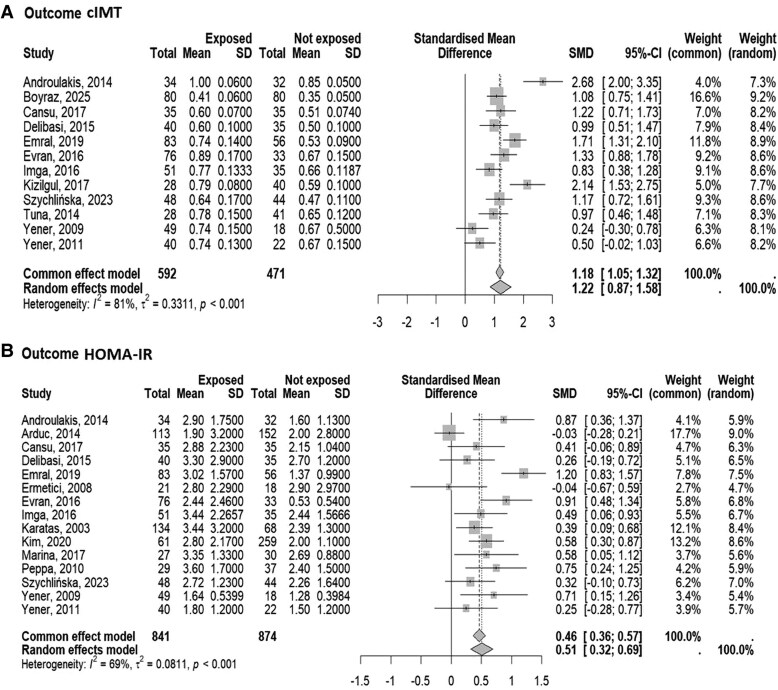
Forest plot illustrating the association between the standardized mean difference in (A) carotid intima-media thickness (cIMT) and (B) Homeostasis Model Assessment for Insulin Resistance (HOMA-IR) between patients with nonfunctioning adrenal tumors and control subjects. The DerSimonian and Laird method, the conventionally used approach for random effects meta-analysis, has been used for calculating the standardized mean difference (SMD) and its 95% CI. The heterogeneity between studies was quantified using *I*^2^ and τ^2^ statistics.

The forest plots illustrating the SMD of cIMT and HOMA-IR with their 95% CI between patients with NFAI compared to controls is shown in Fig. 2. Both the CIMT (Fig. 2A) and HOMA-IR (Fig. 2B) levels in patients with NFAI were higher compared with individuals without adrenal lesion, though with high heterogeneity.

The analysis of funnel plots did not reveal any clear asymmetry, suggesting the absence of publication bias possibly influencing either cIMT or HOMA-IR (supplementary material 55). Likewise, the influence analysis did not show an impact of each study-specific association estimate on the pooled SMD (supplementary material 55).

**Figure 3.**
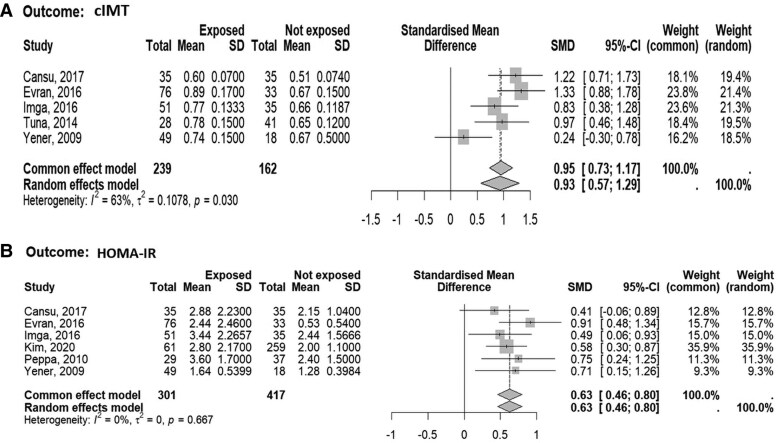
Forest plot illustrating the association between the standardized mean difference in (A) carotid intima-media thickness (cIMT) and (B) Homeostasis Model Assessment for Insulin Resistance (HOMA-IR) between patients with nonfunctioning adrenal tumors and control subjects, including only studies in which the effect of smoking was ruled out (ie, smoking was an exclusion or matching criteria). The DerSimonian and Laird method, the conventionally used approach for random effects meta-analysis, has been used for calculating the standardized mean difference (SMD) and its 95% CI. The heterogeneity between studies was quantified using *I*^2^ and τ^2^ statistics.

The meta-regression analysis showed that in NFAI subjects no associations were present between the SMD of both cIMT and HOMA-IR with mean/median age, proportion of male subjects, and mean/median BMI levels among NFAI subjects. At variance, the CIMT SMD increase but not the HOMA-IR increase was associated with the increase of the percentage of smokers among NFAI subjects (estimate/unit SMD increases on average by 2.145 every percentage point of increase in smokers; 95% CI, 0.43-3.86). However, the forest plots analysis including only studies (5 for cIMT and 6 for HOMA-IR) where the effect of smoking was absent (ie, smokers were excluded or patients were matched for smoking habit) confirmed that cIMT and HOMA-IR levels in patients with NFAI were higher as compared with individuals without adrenal lesion (Fig. 3). In addition, evaluating only studies where patients with type 2 diabetes, hypertension, or dyslipidemia were excluded, did not modify the results on cIMT and HOMA-IR (supplementary materials 55).

### Comparison of AIx, FMD, LVMI, and PWV (Secondary Outcomes) Values Between Patients and Controls

The number of included studies, sample size considered, and the SMD with 95% CI of AIx, FMD, LVMI, and PWV between patients and controls are reported in Table 3.

**Figure 4.**
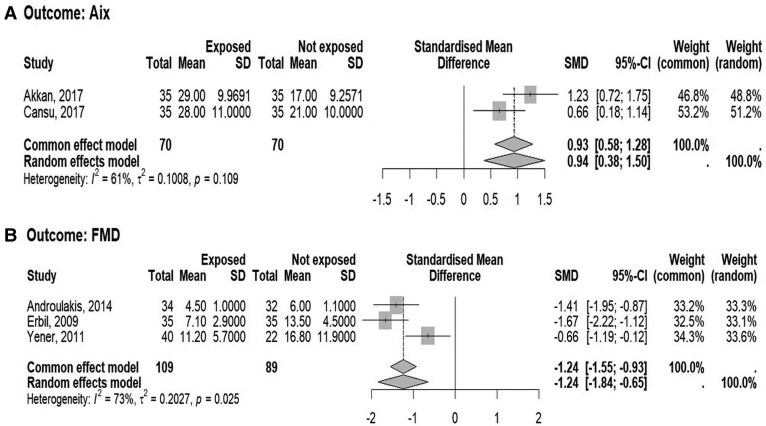
Forest plot illustrating the association between the standardized mean difference in (A) augmentation index (AIx) and (B) flow-mediated dilation (FMD) between patients with nonfunctioning adrenal tumors and control subjects. The DerSimonian and Laird method, the conventionally used approach for random effects meta-analysis, has been used for calculating the standardized mean difference (SMD) and its 95% CI. The heterogeneity between studies was quantified using *I*^2^ and τ^2^ statistics.

**Figure 5.**
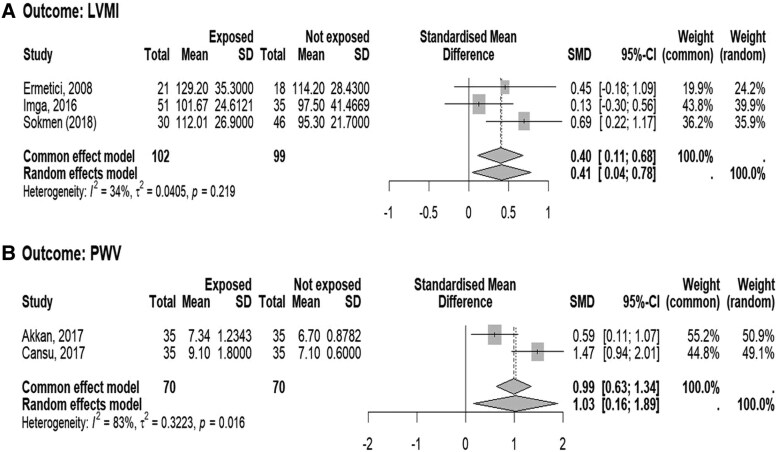
Forest plot illustrating the association between the standardized mean difference in (A) left ventricular mass index (LVMI) and (B) pulse wave velocity (PWV) between patients with nonfunctioning adrenal tumors and control subjects. The DerSimonian and Laird method, the conventionally used approach for random effects meta-analysis, has been used for calculating the standardized mean difference (SMD) and its 95% CI. The heterogeneity between studies was quantified using *I*^2^ and τ^2^ statistics.

The forest plots illustrating the SMD and 95% CI of AIx, FMD, LVMI, and PWV between patients with NFAI compared to controls is shown in Figs. 4A, 4B, 5A, and 5B, respectively. The AIx, LVMI, and PWV levels were higher, whereas FMD levels were lower in patients with NFAI compared with individuals without adrenal lesion, though with high heterogeneity, in particular for FMD and PWV.

Given the low number of the available studies, the analysis of funnel plots, the influence analysis, and the meta-regression analysis have not been done.

## Discussion

In this systematic review and meta-analysis, we evaluated the cardiometabolic risk profile of patients with NFAI compared to individuals without adrenal lesions (controls). We found that patients with NFAI exhibit significantly higher cIMT and increased insulin resistance, as measured by HOMA-IR, relative to controls. Secondary outcomes, including AIx, FMD, LVMI, and PWV, were also significantly different (with AIx, LVMI, and PWV being higher and FMD lower) in patients with NFAI than in controls, although these findings were based on fewer studies and exhibited considerable heterogeneity. Importantly, in all studies the presence of primary aldosteronism has been excluded.

Previous sparse data, summarized in 2 metanalyses, suggested that patients with NFAI have higher prevalence of arterial hypertension, type 2 diabetes, and metabolic syndrome than control subjects without adrenal lesions (8, 9). No meta-analyses were available on the parameters of cardiometabolic disfunction in NFAI patients so far.

The increase in cIMT among patients with NFAI suggests a greater degree of subclinical atherosclerosis, a well-established predictor of cardiovascular risk (19, 20). In addition, the elevated HOMA-IR levels in patients with NFAI suggest the presence of impaired glucose metabolism and insulin resistance, which are known contributors to the development of type 2 diabetes and cardiovascular disease (22). Importantly, these findings were consistent even after controlling for smoking status, age, sex, and BMI, indicating that the presence of NFAI may independently contribute to cardiometabolic risk. The observed elevations in AIx, FMD, LVMI, and PWV, although requiring further confirmation, align with the notion that NFAI is associated with vascular stiffness, endothelial dysfunction, and cardiac remodelling (16-18, 47).

In particular, endothelial dysfunction, as evaluated in vivo and noninvasively through FMD, represents the earliest phase of the arteriosclerotic and atherosclerotic process. That it is more impaired (ie, lower FMD values) in patients with NFAI suggests the presence of very early and detrimental vascular effects. In the subsequent phases, arteries progressively stiffen, as assessed through PWV, leading to an increased speed of the reflected pressure wave, which can be evaluated through the AIx. Finally, the increased vascular resistance against which the heart must pump results in cardiac hypertrophy, assessed by a higher LVMI. The results of our review confirm that NFAI is associated with all evaluated parameters, from insulin resistance to LVMI, thus spanning the entire spectrum of the atherosclerotic process (56, 57).

From a clinical perspective, these results challenge the current classification of NFAI as entirely nonfunctioning based on the existing F-1mgDST threshold of 1.8 µg/dL (50 nmol/L). Subtle cortisol secretion below the threshold of detection by conventional testing may nonetheless exert deleterious effects on cardiovascular and metabolic health. This underscores the need for heightened clinical vigilance and possibly a reevaluation of biochemical criteria used to define cortisol autonomy in patients with adrenal incidentalomas (58). The knowledge that at least some patients with NFAI may be at higher risk of cardiometabolic consequences may foster an early identification of patients with increased cardiovascular risk and guide tailored interventions, including closer monitoring, lifestyle modifications, medical treatment of risk factors, and potentially surgical treatment, which has shown benefits in patients with MACS (6, 59).

Despite its strengths, the study has several limitations that shape the path for future research. First, considerable heterogeneity existed, particularly in the analysis of secondary endpoints like AIx and PWV, which were supported by only 2 to 3 studies each. This limited the statistical power and prevented detailed analyses. Moreover, even the studies’ design was partially heterogeneous because data collection was prospective and retrospective in 13 and 2 studies, respectively, whereas the study design was not reported in 6 studies. The inclusion of more high-quality studies, or even an individual patient data meta-analysis, could improve the robustness of future findings.

Second, all included studies were cross-sectional in nature, limiting causal interpretation. While associations between NFAI and cardiometabolic derangements are clearly demonstrated, it remains unknown whether these lead to increased rates of cardiovascular events over time. Importantly, relying on surrogate markers instead of hard cardiovascular endpoints (eg, myocardial infarction, stroke, cardiovascular death) limits the clinical applicability of the present findings. These surrogates, though associated with cardiovascular risk, have varying predictive accuracy and may be influenced by confounding factors such as age, comorbidities, and treatment status. Notably, HOMA-IR and cIMT, though correlated in some contexts, measure distinct aspects of metabolic and vascular health and their predictive value on hard end-points in low-risk or asymptomatic populations remains debated. Indeed, even though cIMT is considered a marker of subclinical atherosclerosis, its accuracy in predicting hard cardiovascular outcomes is debated, with some data showing that they provide low incremental predictive value beyond traditional risk scores (60, 61). In addition, it should be underlined that most evidence on the utility of these markers is observational and only few robust data support clinical decision-making based on these markers, which explains why none of these surrogate endpoints are universally endorsed by guidelines for routine use in risk assessment or treatment monitoring (62). Furthermore, the surrogate endpoints assessed, ranged from structural (eg, cIMT), to functional (eg, FMD, AIx, PWV), to metabolic (eg, HOMA-IR), thus mirroring different aspects of the cardiovascular disease continuum. Importantly, because some of these parameters may influence each other (eg, HOMA-IR and cIMT) their individual predictive value on hard end-points is often poor.

On the other hand, HOMA-IR, though useful in identifying insulin resistance, is influenced by glycemic fluctuations and pharmacologic treatment, particularly in those with diabetes, and may not reliably reflect long-term metabolic risk once diabetes is diagnosed and treated (63, 64). However, when analyzing only studies in which patients with type 2 diabetes were excluded, the results were confirmed. This helps reducing the possible influence of type 2 diabetes on the interpretation of the effect of NFAI on cIMT and HOMA-IR (supplementary materials 55).

Third, in the absence of patient's individual data, the influence of baseline clinical variables such as blood pressure, lipid profiles, and medication use, known determinants of cardiovascular risk could not be excluded. As highlighted by the INTERHEART study, >90% of cardiovascular events can be explained by traditional risk factors such as smoking, hypertension, and dyslipidemia (65). The absence of this information in some included studies complicates the interpretation of whether NFAI contributes independently to cardiometabolic risk, or whether the observed alterations in surrogate markers reflect underlying, unadjusted risk profiles. However, when considering only studies excluding patients with smoking habit or matched for smoking habit (Fig. 3) or those matched for hypertension or dyslipidemia (supplementary materials 55), the results were confirmed, reinforcing the reliability of the present results. On the other hand, the possible increase in cardiovascular risk in patients with NFAI is still a matter of debate, possibly because of the small sample size of the available studies (9). Longitudinal studies capturing incident cardiovascular events will be critical to establish whether these surrogate alterations translate into meaningful clinical outcomes in patients with NFAI.

Additional concerns include residual confounding from unmeasured lifestyle factors such as diet, exercise, and socioeconomic status. Although some studies adjusted for common risk variables, many did not account for these key influencers. Also noteworthy is the role of smoking, which was found to mediate the relationship between NFAI and increased cIMT in some analyses. This raises the question of whether lifestyle factors might amplify or mask the metabolic impact of these adrenal lesions. Future primary studies to include more comprehensive lifestyle and socioeconomic variables (eg, diet, physical activity, medication use, smoking) are needed to allow for deeper adjustment in pooled analyses.

Fourth, there was a striking lack of geographic diversity among the study populations. Of the 21 studies analyzed, 20 were European and only 1 came from East Asia. This homogeneity limits the generalizability of the findings to more diverse ethnic and regional populations, especially those in North America, Africa, and Latin America. Given the known differences in cardiovascular risk across ethnic groups, future research should prioritize more inclusive study designs.

Finally, it is also possible to speculate that the increase in F-1mgDST levels could be secondary to comorbidities rather than specifically associated with the presence of AI, especially in studies including patients living with diabetes (66). In addition, it cannot be excluded that individuals undergoing repeated imaging procedures at radiology facilities (eg, patients with NFAI) are more likely to have underlying medical conditions, and therefore a higher likelihood of disease, compared to those who undergo imaging only once.

Notwithstanding these limitations, this study raises an important issue regarding terminology. The label “nonfunctioning” may be misleading, as subtle hormonal activity might still influences cardiometabolic health even when standard biochemical thresholds are not exceeded. Alternative terms, such as “adrenal incidentaloma with low-grade autonomous cortisol secretion,” may better reflect the emerging understanding of these lesions. Future studies should explore more sensitive hormonal assays and refined diagnostic thresholds to better stratify patient risk.

## Data Availability

Some or all datasets generated and/or analyzed during the current study are not publicly available but are available from the corresponding author on reasonable request.

## Abbreviations

AI: adrenal incidentaloma
Aix: augmentation index
BMI: body mass index
cIMT: carotid intima-media thickness
F-1mgDST: 1 mg overnight dexamethasone suppression test
FMD: flow-mediated dilation
HOMA-IR: Homeostasis Model Assessment for Insulin Resistance
LVMI: left ventricular mass index
MACS: mild autonomous cortisol secretion
NFAI: nonfunctioning adrenal incidentaloma
PWV: pulse wave velocity
SMD: standardized mean difference
